# Blockade of LAG-3 Immune Checkpoint Combined With Therapeutic Vaccination Restore the Function of Tissue-Resident Anti-viral CD8^+^ T Cells and Protect Against Recurrent Ocular Herpes Simplex Infection and Disease

**DOI:** 10.3389/fimmu.2018.02922

**Published:** 2018-12-17

**Authors:** Soumyabrata Roy, Pierre-Grégoire Coulon, Ruchi Srivastava, Hawa Vahed, Grace J. Kim, Sager S. Walia, Taikun Yamada, Mona A. Fouladi, Vincent T. Ly, Lbachir BenMohamed

**Affiliations:** ^1^Laboratory of Cellular and Molecular Immunology, School of Medicine, Gavin Herbert Eye Institute, University of California, Irvine, Irvine, CA, United States; ^2^Department of Molecular Biology and Biochemistry, University of California, Irvine, Irvine, CA, United States; ^3^Institute for Immunology, School of Medicine, University of California, Irvine, Irvine, CA, United States

**Keywords:** herpes simplex type 1, CD8^+^ T cells, LAG-3, immune check point, recurrent, therapeutic, animal model, humans

## Abstract

Recurrent viral diseases often occur after the viruses evade the hosts' immune system, by inducing exhaustion of antiviral T cells. In the present study, we found that functionally exhausted herpes simplex virus type 1 (HSV-1) -specific CD8^+^ T cells, with elevated expression of lymphocyte activation gene-3 (LAG-3), an immune checkpoint receptor that promotes T cell exhaustion, were frequent in symptomatic (SYMP) patients with a history of numerous episodes of recurrent corneal herpetic disease. Similarly, following UV-B induced virus reactivation from latency the symptomatic wild-type (WT) B6 mice that developed increase virus shedding and severe recurrent corneal herpetic disease had more exhausted HSV-specific LAG-3^+^CD8^+^ T cells in both trigeminal ganglia (TG) and cornea. Moreover, a therapeutic blockade of LAG-3 immune checkpoint with antagonist antibodies combined with a therapeutic immunization with gB_498−505_ peptide immunodominant epitope of latently infected B6 mice significantly restored the quality and quantity of functional HSV-1 gB_498−505_ specific CD8^+^ T cells in both TG and cornea and protected against UV-B induced recurrent corneal herpes infection and disease. In contrast to dysfunctional HSV-specific CD8^+^ T cells from WT B6 mice, more functional HSV-specific CD8^+^ T cells were detected in LAG-3^−/−^ deficient mice and were associated with less UV-B induced recurrent corneal herpetic disease. Thus, the LAG-3 pathway plays a fundamental role in ocular herpes T cell immunopathology and provides an important immune checkpoint target that can synergizes with T cell-based therapeutic vaccines against symptomatic recurrent ocular herpes.

## Introduction

A staggering 3.72 billion individuals worldwide are infected with herpes simplex virus type 1 (HSV-1), a prevalent human viral pathogen (1–3). Herpes infection and reactivation cause complications which range from mild, such as cold sores and genital lesion, to grave, such as permanent brain damage from encephalitis in adults and neonates, and blinding recurrent corneal herpetic disease (4). After a primary acute infection of the cornea, HSV-1 travels up sensory neurons to the trigeminal ganglia (TG) where it establishes lifelong latency in its host (5–9). Potentially blinding keratitis occurring from recurrent corneal herpetic disease results from the reactivation of latent virus from neurons of the TG, anterograde transportation to nerve termini, and re-infection of the cornea (8, 9).

Controlling the establishment of HSV-1 latency and preventing reactivation from TG involves dynamic crosstalk between the virus and CD8^+^ T cells within the latently infected TG microenvironment (5, 6, 8–10). However, the molecular mechanisms by which such interactions occur remain to be fully elucidated. HSV-specific CD8^+^ T cells are selectively activated and retained in the tissues of latently infected TG (6, 8, 9). On one hand, HSV-specific CD8^+^ T cells can significantly reduce reactivation in TG explant from latently infected mice (5, 9), apparently by interfering with virus replication and spread following the initial molecular events of reactivation (5, 8, 9). On the other hand, HSV-1 can manage to reactivate in the face of an often-sizable pool of virus-specific CD8^+^ T cells in the TG, apparently by interfering with the quality and quantity of CD8^+^ T cells that reside in the TG (6, 9, 11). Thus, the virus appears to keep CD8^+^ T cells “in check” using among several mechanisms, functional impairment of T cells (i.e., exhaustion), which is usually the result of prolonged exposure of T cell to high levels of viral antigens, as occurs during productive chronic infections (12, 13). Many viruses, including HSV-1, appear to reactivate from latency and sustain their productive infection by inducing functional exhaustion of antiviral CD8^+^ T cells (10, 12, 14–17).

Total or partial loss of T cell function occurs following repetitive HSV-1 latent/reactivation cycles, sporadic events that occur in latently infected trigeminal ganglia (TG) (10, 18, 19). T cell dysfunction requires two signals: (1) T cell receptors (TCR) engaged by MHC presenting an HSV epitope (16); and (2) T cell co-inhibitory receptors engaged by their ligands expressed on infected cells (e.g., infected sensory neurons of TG) (10, 20). When T cell dysfunction develops under conditions of repetitive exposure to viral antigens it is called exhaustion [reviewed in (21)]. This is usually linked with the expression of a long list of T cell co-inhibitory receptors including: programmed death-1 (PD-1), T cell immunoglobulin mucin-(TIM)-3, lymphocyte activation gene-3 (LAG-3, also known as CD223), T cell immunoreceptor with Ig and ITIM domains (TIGIT), P-selectin glycoprotein ligand-1 (PSGL-1), 2B4 (also known as CD244), glucocorticoid-induced TNFR-related protein (GITR, also known as TNFRSF18), CD160, cytotoxic T-lymphocyte-associated protein 4 (CTLA-4, also known as CD152), B- and T-lymphocyte attenuator (BTLA also known as CD272), and V-domain immunoglobulin suppressor of T cell activation (VISTA) [reviewed in (16, 22)]. In humans, sporadic molecular reactivations of latent HSV-1 from sensory neurons of the TG is accompanied by chronic CD8^+^ T cell infiltrates (23–27). The cellular and molecular immune mechanisms that control the HSV-1 latency-reactivation cycle remain to be fully elucidated. Nevertheless, at least a portion of these virus reactivations in the TG appears to be controlled by CD8^+^ T cell-mediated mechanisms (8, 10, 28). Many of these ganglia-resident CD8^+^ T cells express PD-1 (25). However, there is not much information: (*i*) on the exhaustion states of HSV-specific CD8^+^ T cells that reside in the TG of HSV-1 seropositive individuals; nor (*i*) on the phenotypic and functional exhaustion characteristics of HSV-specific CD8^+^ T cells from symptomatic (SYMP) individuals (who develop frequent, recurrent herpetic disease) and asymptomatic (ASYMP) individuals (who never experience any recurrent herpetic disease despite being infected). Based on these collective observations, we hypothesized that: (*i*) HSV-1 latently infected TG, with spontaneous or UV-B induced sporadic virus reactivation, would harbor CD8^+^ T cells that express at least some of the T cell co-inhibitory receptors above and exhibit functional exhaustion; and (*ii*) therapeutic blockade of the highly expressed T cell inhibitory receptors, to restore the function of TG-resident anti-viral CD8^+^ T cells combined with a therapeutic vaccination to further boost the number and the function of HSV-specific CD8^+^ T cells that reside in TG would markedly improve clinical outcomes and protect against recurrent corneal herpes infection and disease.

In the present study, we tested the above hypotheses by: (*i*) Comparing phenotypic and functional exhaustion of peripheral blood-derived HSV-specific CD8^+^ T cells from SYMP and ASYMP individuals; and (*ii*) Studying phenotypic and functional exhaustion of cornea and TG-derived HSV-specific CD8^+^ T cells using our established mouse model of recurrent ocular herpes. In this model, UV-B irradiation of the cornea of latently infected B6 mice induces HSV-1 reactivation from latently infected TG, as measured by shedding of reactivated virus in tears, in turn leading to recurrent herpetic corneal disease (29, 30). We found that: (*i*) Both PD-1 and LAG-3 co-inhibitory receptors were expressed at significantly higher levels on HSV-specific CD8^+^ T cells from SYMP individuals, with severe recurrent corneal disease, compared to ASYMP individuals with no disease; (*ii*) Higher prevalence of HSV-specific LAG-3^+^CD8^+^ T cells and PD-1^+^CD8^+^ T cells were present in SYMP individuals compared to ASYMP individuals; (*iii*) In the B6 mouse model of recurrent ocular herpes, following UV-B induced reactivation, most effector CD8^+^ T cells from the cornea and TG expressed higher levels of LAG-3 and PD-1; (*iv*) This phenotype correlated with functional exhaustion of HSV-specific CD8^+^ T cells and with increased virus reactivation, as measured by shedding of reactivated virus in tears, and severe recurrent cornea herpetic disease; and (*v*) Blockade of LAG-3 pathway combined with therapeutic immunization of latently infected B6- albino mice reversed the exhaustion of HSV-specific CD8^+^ T cells, in both TG and cornea, associated with protection against UV-B induced recurrent corneal herpes infection and disease. Overall, our findings suggest that: (*i*) Besides PD-1, the LAG-3 pathway plays a fundamental role in controlling herpes T cell immunity; (*ii*) Blockade of the LAG-3 pathway provides an important immune checkpoint that can synergize with T cell-based therapeutic herpes vaccines to protect against recurrent ocular herpes.

## Materials and Methods

### Human Study Population

All clinical investigations in this study were conducted according to the Declaration of Helsinki. All subjects were enrolled at the University of California, Irvine under approved Institutional Review Board-approved protocols (IRB#2003-3111 and IRB#2009-6963). Written informed consent was received from all participants prior to inclusion in the study.

During the last 15 years (i.e., January 2003 to July 2018), we have screened 875 individuals for HSV-1 and HSV-2 seropositivity. Patients were segregated into SYMP and ASYMP individuals based on the inclusion criteria as previously described (2, 32–33). Among the large cohort of SYMP and ASYMP individuals, 16 HLA-A^*^02:01 positive patients (8 ASYMP and 8 SYMP) were enrolled in this study (Table 1). SYMP and ASYMP groups were matched for age, gender, serological status, and race. The HLA-A2 status was confirmed by PBMC staining with 2 μl of anti-HLA-A2 mAb (clone BB7.2; BD Pharmingen Inc., San Diego, CA), at 4°C for 30 min. The cells were washed and analyzed by flow cytometry using a LSRII (Becton Dickinson, Franklin Lakes, NJ). The acquired data were analyzed with FlowJo software (BD Biosciences, San Jose, CA).

**Table 1:** Cohorts of HLA-A^*^02:01 positive, HSV seropositive symptomatic and asymptomatic individuals enrolled in the study.

**Subject-level characteristic**	**All subjects (*n* = 39)**
**Gender [no. (%)]:**	
Female	15 (51%)
Male	14 (49%)
**Race [no. (%)]**	
Caucasian	19 (66%)
Non-Caucasian	10 (34%)
Age [median (range) years]	39 (21–67 years)
**HSV status [no. (%)]**	
HSV-1 seropositive	29 (100%)
HSV-2 seropositive	0 (0%)
HSV-1 and -2 seropositive	0 (0%)
HSV seronegative	10 (100%)
**HLA [no. (%)]**	
HLA-A^*^02:01 positive	24 (83%)
HLA-A^*^02:01 negative	5 (17%)
**Herpes disease status [no. (%)]**	
Asymptomatic (ASYMP)	19 (66%)
Symptomatic (SYMP)	10 (34%)

*Definition of symptomatic and asymptomatic individuals are detailed in Materials and Methods*.

### Human Peripheral Blood Mononuclear Cells (PBMC) Isolation

Individuals (negative for HIV, HBV, and with or without any HSV infection history) were recruited at the UC Irvine Institute for Clinical and Translational Science (ICTS). Between 40 and 100 mL of blood was drawn into Vacutainer® Tubes (Becton Dickinson). The serum was isolated and stored at −80°C for the detection of anti-HSV-1 and HSV-2 antibodies, as we have previously described (31). PBMCs were isolated by gradient centrifugation using leukocyte separation medium (Life Sciences, Tewksbury, MA). The cells were then washed in PBS and re-suspended in complete culture medium consisting of RPMI1640, 10% FBS (Bio-Products, Woodland, CA) supplemented with 1x penicillin/streptomycin/L-glutamine, 1x sodium pyruvate, 1x non-essential amino acids, and 50 μM of 2-mercaptoethanol (Life Technologies, Rockville, MD). Freshly isolated PBMCs were also cryo-preserved in 90% FCS and 10% DMSO in liquid nitrogen for future testing.

### Human T Cells Flow Cytometry Assays

The following anti-human antibodies were used for the flow cytometry assays: CD3 A700 (clone SK7; BioLegend, San Diego, CA), CD8 PE-Cy7 (clone SK1; BioLegend) PD-1 FITC (clone EH12.2H7; BioLegend), LAG-3 PerCPCy5.5 (clone 11C3C65; BioLegend), For the surface stain, mAbs against cell markers were added to a total of 1 × 10^6^ cells in 1X PBS containing 1% FBS and 0.1% sodium azide (FACS buffer) for 45 min at 4°C. After washing twice with FACS buffer, cells were fixed in PBS containing 2% paraformaldehyde (Sigma-Aldrich, St. Louis, MO). For each sample, 100,000 total events were acquired on the BD LSRII. Ab capture beads (BD Biosciences) were used as individual compensation tubes for each fluorophore in the experiment. To define positive and negative populations, we used fluorescence minus controls for each fluorophore. Furthermore, we optimized gating by examining known negative cell populations for background expression levels similar to that used in our previous work (7). Briefly, we gated single cells, dump cells, viable cells (Aqua Blue), lymphocytes, CD3^+^ cells, and CD8^+^ cells before finally gating human epitope-specific CD8^+^ T cells using HSV-specific tetramers (Figure S1). Data analysis was performed using FlowJo software (BD Biosciences, San Jose, CA). Statistical analyses were done using GraphPad Prism version 5 (La Jolla, CA).

### Tetramer/VP11/12 Peptide Staining

Fresh PBMCs were analyzed for the frequency of CD8^+^ T cells recognizing the VP11/12 peptide/tetramer complexes, as we previously described (32–35). The cells were incubated with VP11/12 peptide/tetramer complex for 30–45 min at 37°C. The cell preparations were then washed with FACS buffer and stained with FITC-conjugated anti-human CD8 mAb (BD Pharmingen). The cells were then washed and fixed with 1% paraformaldehyde in PBS and subsequently acquired on a BD LSRII. Data were analyzed using FlowJo version 9.5.6 (Tree Star).

### Mice

Female B6(Cg)-*Tyr*^*c*−2*J*^/J, or B6-albino mice and LAG-3-deficient mice (LAG-3^−/−^ mice) (6 to 8 weeks old; on the C57BL/6 background) and female C57BL/6 (B6) wild-type (WT) mice (6 to 8 weeks old) were purchased from the Jackson Laboratory (Bar Harbor, ME). Animal studies were performed conforming to the *Guide for the Care and Use of Laboratory Animals* (28). Experiments were conducted with the approval of the Institutional Care and Use Committee of University of California Irvine (Irvine, CA).

### Virus Production and the Ocular Challenge of Mice With HSV-1

HSV-1 (strain McKrae) was grown and tittered on rabbit skin (RS) cells as described previously (20–22). All types of mice were ocularly infected with either with 2 × 10^5^ PFU (acute phase studies) or 1 × 10^6^ PFU (reactivation studies) of strain McKrae via eye drops. Following ocular infection, mice were monitored for ocular herpes virus infection and disease.

### Immunization With Immunodominant gB_498−505_ Peptide SSIEFARL

Age-matched female mice of each type were assorted in various groups (*n* = 10/group). As per the experimental plan, groups of mice were immunized subcutaneously (s.c.) with the immunodominant gB_498−505_ peptide SSIEFARL delivered with the promiscuous CD4+ T helper (Th) epitope PADRE and CpG1826 adjuvant on day 18 post-infection (PI) followed by a booster dose on day 25 PI. All immunizations were carried out with 100 uM of each peptide.

### UV-B Induced Reactivation of HSV-1 From Latency in Mice

**Thirty-five** days post-infection, when latency was fully established, reactivation of latent HSV-1 infection was induced following UV-B irradiation in all groups of mice (30). TM20 Chromato-Vu transilluminator (UVP, San Gabriel, CA), which emits UV-B at a peak wavelength of 302 nm was used for the purpose. Anesthetized [Intraperitoneal (IP) injection of ketamine/xylazine mouse cocktail 0.1 mL/20 g mouse containing 87.5 mg/kg ketamine and 12.5 mg/kg xylazine] mice were placed on the transilluminator, and each mouse was positioned on a piece of cardboard containing a hole the same size as the mouse's eye. This allowed just the eyes to be irradiated by the UV-B source. Each eye was irradiated with 250 mJ/cm^2^ of UV-B light (60-s exposure on the transilluminator).

### PD-1 and LAG-3 Blockade in Mice

Anti-PD-1 mAb (RMPI-14) and anti-LAG-3 mAb (C9B7W) were purchased from BioXcell (West Lebanon, NH). For acute phase studies, WT B6 mice were ocularly infected with 2 × 10^5^ PFU of strain McKrae and treated on day 3, 5, and 7 with IP injection of 200 μg of anti-PD-1 mAb or anti-LAG-3 mAb during the acute phase. For reactivation studies, in some designated groups, UV-B irradiation was performed on day 35 and subsequently treated on day 37, 39, and 41 with IP injection of 200 μg of anti-LAG-3 mAb.

### Monitoring of Ocular Herpes Infection and Disease in Mice

Virus shedding during the acute phase and that induced by UV-B irradiation was quantified in eye swabs collected every day during the acute phase and post-UV-B irradiation (up to day 8). Eyes were swabbed using moist type 1 calcium alginate swabs and frozen at −80°C until titrated on RS cell monolayers, as described previously (30–34). Animals were examined for signs of recurrent corneal herpetic disease by slit lamp camera (Kowa American Corporation, Torrance CA 90502), for 30 days post UV-B radiation; this was performed by investigators who were blinded to the treatment regimen of the mice and scored according to a standard 0–4 scale (0 = no disease; 1 = 25%; 2 = 50%; 3 = 75%; 4 = 100%) as previously described (30, 31). Total disease score of each day in each group of mice till 30-days post-UV-B exposure was noted. Cumulative graphs of eye disease were generated by dividing the total score of each day per group of mice by total number of eyes in each group and adding the value to that obtained in the succeeding day and continuing till day 30 post-UV-B. Similarly, cumulative graphs of the number of eyes showing recurrent keratitis were done by dividing the total number of eyes showing disease per group of mice (irrespective of disease severity) by the total number of eyes in each group and adding the value to that obtained in the following day and continuing till 30-days post-UV-B. Average of the total score of each group for each of the 30 days post UV-B was calculated by dividing the total score of each day by the total number of eyes in each group.

### Isolation of Lymphocytes

Mice from all groups were euthanized, and the spleen, cornea, and TG were individually harvested. Cornea and TG tissues were digested in complete medium containing 2.5-mg/ml collagenase type IV (Sigma Chemical Co., St. Louis, MO). Digestion was accomplished by incubation at 37°C with shaking for 30 min. After digestion, tissues and cells were filtered through a sterile gauze mesh and washed with RPMI 1640 medium. Spleen homogenates were prepared by pressing the tissue through a sterile mesh screen into 10 ml of PBS under aseptic conditions. Single-cell suspensions thus prepared from spleen, TG and cornea were analyzed using flow cytometry (Figure S1).

### Mice Flow Cytometry Analysis

The following anti-mouse antibodies were used: CD3 FITC (clone17A2; Biolegend), CD8 PerCP (clone 53-6.7; BD), CD107a FITC (clone 1D4B; BD), CD107b FITC (clone Ha1/29; BD), IFN-γ-PE (clone XMG1.2; BioLegend), and Ki-67 PE/Cy7 (clone 16A8; BioLegend). Both surface and intracellular staining were performed similarly to the human study as described above.

### Tetramer/gB_498−505_ Staining

Cells harvested from spleen, TG and cornea were analyzed for the frequency of CD8^+^T cells recognizing gB_498−505_ peptide tetramer complex similarly to the human study as aforementioned_._

### Statistical Analysis

Data for each assay were compared by ANOVA and Student's *t*-test using GraphPad Prism version 5 (La Jolla, CA). Differences between the groups were identified by ANOVA and multiple comparison procedures, as we previously described (33, 34). Data are expressed as the mean ± SD. Results were considered statistically significant at *P* value of ≤ 0.05.

## Results

### HSV-Specific CD8^+^ T Cells, With Elevated Expression of PD-1 and LAG-3, Are Frequent in Symptomatic Patients With Recurrent Herpetic Disease

The characteristics of the symptomatic (SYMP) and asymptomatic (ASYMP) study population used in this present study, with respect to gender, age, HLA-A^*^02:01 frequency distribution, HSV-1/HSV-2 seropositivity and status of ocular and genital herpetic diseases are presented in Table 1 and detailed in the *Materials* and *Methods section*. Since HSV-1 is the main cause of ocular herpes, only individuals who are HSV-1 seropositive and HSV-2 seronegative were enrolled in the present study. HSV-1 seropositive individuals were divided into two groups: (*i*) ten HLA-A^*^02:01 positive, HSV-1-infected ASYMP individuals who have never had any clinically detectable herpes disease; and (*ii*) ten HLA-A^*^02:01 positive HSV-1-infected SYMP individuals with a history of numerous episodes of well-documented recurrent clinical herpes diseases, such as herpetic lid lesions, herpetic conjunctivitis, dendritic or geographic keratitis, stromal keratitis, and iritis consistent with rHSK, with one or more episodes per year for the past 5 years. Only SYMP patients who were not on Acyclovir or other anti-viral or anti-inflammatory drug treatments at the time of blood sample collections were enrolled. One patient had over two severe recurrent episodes during the last 10 years that necessitated multiple corneal transplantations.

**Figure 1:**
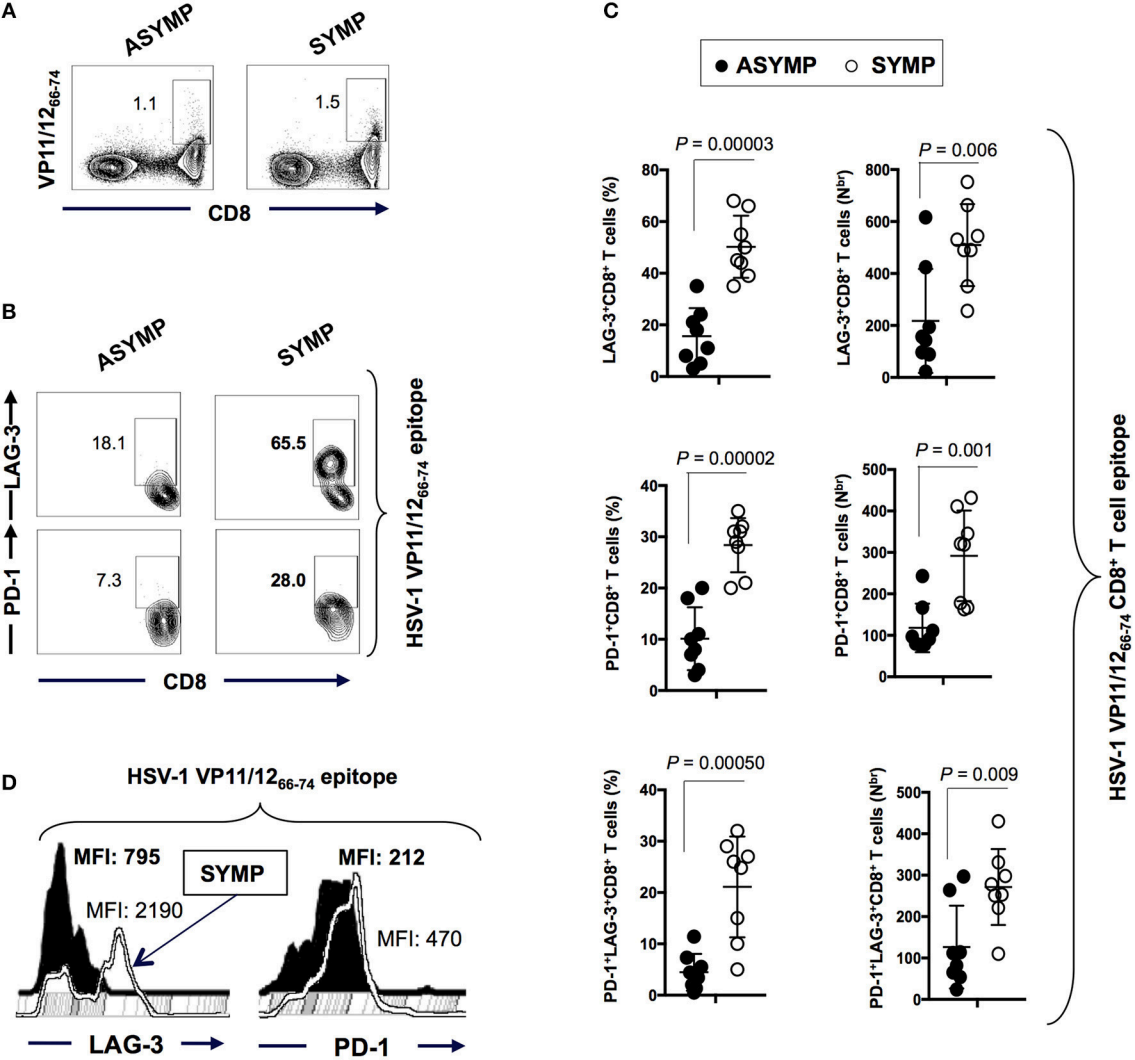
Frequency of HSV-1 VP11/12_66−74_ epitope-specific LAG-3^+^CD8^+^T cells and PD-1^+^CD8^+^T cells in ASYMP vs. SYMP individuals. **(A)** Representative FACS plot of the frequencies of HSV-1 VP11/12_66−74_ tetramer-specific CD8^+^ T cells in ASYMP vs. SYMP individuals. **(B)** Representative FACS plot of the frequencies of LAG-3^+^CD8^+^T cells and PD-1^+^CD8^+^T cells in ASYMP and SYMP individuals. **(C)** Average percentages (*left panels*) and the absolute number (*right panels*) of HSV-1 VP11/12_66−74_ tetramer-specific PD-1^+^ CD8^+^ T cells, LAG-3^+^ CD8^+^ T cells and PD-1^+^LAG-3^+^CD8^+^ T cells in ASYMP and SYMP individuals. **(D)** level of expression of LAG-3 and PD-1 receptors onCD8^+^ T cells from ASYMP vs. SYMP individuals, depicted as Mean fluorescent intensity (MFI). Results are representative of two independent experiments in each individual. The indicated *P*-values, calculated using the unpaired *t*-test, show statistical significance between SYMP and ASYMP individuals.

We first sought to determine whether there is any differential frequency of HSV-specific CD8^+^ T cells expressing exhaustion markers PD-1 and LAG-3 between SYMP and ASYMP individuals. Blood-derived HSV-1 VP11/12_66−74_ epitope specific CD8^+^ T cells from SYMP and ASYMP (*n* = 8, each) individuals were analyzed by flow cytometry for the expression of LAG-3 and PD-1. A tetramer specific to the immunodominant VP11/12_66−74_ epitope was used to decipher the expression of LAG-3 and PD-1 uniquely on HSV-specific T cell (instead of bulk CD8^+^ T cells). As shown in Figure 1A, there were no observed differences in the frequency of VP11/12_66−74_ epitope-specific CD8^+^T cells between ASYMP (1.1%) and SYMP (1.5%) individuals. However, VP11/12_66−74_ epitope-specific LAG-3^+^CD8^+^T cells and PD-1^+^CD8^+^T cells appeared to be more frequent in SYMP compared to ASYMP individuals (Figures 1B,C). Moreover, as shown in Figure 1D, elevated expression levels of LAG-3 and PD-1 were detected in VP11/12_66−74_ epitope-specific CD8^+^T cells of SYMP patients compared to ASYMP healthy individuals, as depicted by a significant difference in mean fluorescent intensity (MFI) of LAG-3 and PD-1 expression.

Altogether these results indicate that both PD-1 and LAG-3 markers of exhaustion are highly expressed in HSV-specific CD8^+^ T cells from SYMP patients that are clinically diagnosed with the repetitive recurrent ocular herpetic disease. This data is in agreement with the functional impairment of VP11/12_66−74_-specific CD8^+^ T cells we have previously reported on in SYMP individuals (2). Since LAG-3 and PD-1 markers are strong determinants of functional exhaustion, this denotes that exhaustion of antigen-specific CD8^+^ T cells in SYMP individuals may be a potential cause of the suboptimal immunity, often associated with symptomatic shedding.

Because of ethical and practical complexities in obtaining cornea- and trigeminal ganglia- (TG) derived CD8^+^ T cells in humans, we were limited to using blood-derived CD8^+^ T cells in humans. However, the phenotype and function of human blood-derived CD8^+^ T cells may not reflect tissue-resident CD8^+^ T cells. For these reasons, the remainder of this study utilized our established mouse model of acute and UV-B induced recurrent ocular herpes to determine the phenotypic and functional exhaustion of TG- and cornea-resident CD8^+^ T cells and their association with acute and recurrent ocular herpes. Since our results above on human blood-derived CD8^+^ T cells suggests high frequencies of HSV specific CD8^+^ T cells expressing LAG-3 and PD-1 in SYMP individuals; next, we determined the kinetics of LAG-3 and PD-1 expression in cornea and TG following HSV-1 infection in mice.

### Increased Frequency and Number of HSV Specific LAG-3^+^CD8^+^ T Cells in the Cornea and TG of Ocular Herpes Infected Mice

**Figure 2:**
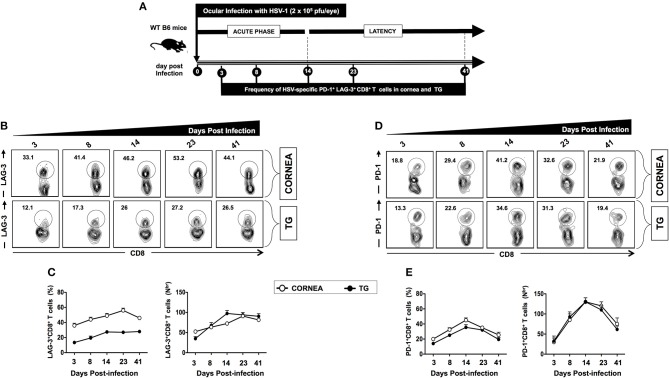
Kinetics of LAG-3 and PD-1 expression on HSV-specific CD8+ T cells following ocular herpes virus infection. **(A)** Schematic representation of the timeline for HSV-1 infection and assays on phenotypic exhaustion of HSV-specific CD8^+^ T cells in WT B6 mice (6–8 weeks old, *n* = 40), following ocular infection on day 0 with 2 × 10^5^ pfu of HSV-1 strain McKrae, as described in *Materials* and *Methods*. A group of 10 mice were sacrificed on days 3, 8, 14 (acute phase), 23, and 41 (latent phase) post-infection (PI). Subsequently, cornea and TG were harvested, and the frequencies of the HSV specific (gB_498−505_) CD8^+^ T cells expressing LAG-3 and PD-1 exhaustion markers were determined by FACS. **(B)** Representative FACS plot and **(C)** average percentages and absolute numbers of HSV-specific CD8^+^ T cells expressing LAG-3 are detected in cornea and TG at different time points. **(D)** Representative FACS plot, and **(E)** average percentages and absolute numbers of HSV-specific CD8^+^ T cells expressing PD-1 detected in cornea and TG at different time points. The results are representative of two independent experiments.

A group of 40 mice were infected with 2 × 10^5^ pfu of HSV-1 strain McKrae. Mice (*n* = 10) were sacrificed during acute and latent phases at five different time points (i.e., days 3, 8, 14, 23, and 41). Cornea and TG were harvested, and the frequencies of HSV specific (gB_498−505_) CD8^+^ T cells expressing LAG-3 and PD-1 exhaustion markers were analyzed by FACS. The frequencies of HSV specific (gB_498−505_) CD8^+^ T cells expressing LAG-3 appeared to increase starting on day 3 during acute infection in both cornea (33.1%) and TG (12.1%) (Figures 2A–**E**). The highest frequencies of HSV specific (gB_498−505_) CD8^+^ T cells expressing LAG-3 were detected on day 23 during latency in both cornea (53.2%) and TG (27.2%), and those seem to persist until day 41 of latency (Figures 2B,C). Similarly, the frequencies of HSV specific (gB_498−505_) CD8^+^ T cells expressing PD-1 increased in both the cornea and TG starting on day 3 during acute infection in both cornea (18.8%) and TG (13.3%) (Figures 2D,E). Further heightened expression of PD-1 was observed late in acute phase on day 14 in both cornea (41.2%) and TG (34.6%) and gradually diminished by day 41 during latency (Figures 2D,E). Intriguingly, high frequencies of LAG-3^+^CD8^+^ T cells, but not of PD-1^+^CD8^+^ T cells, were found in the cornea. In contrast, similar frequencies of LAG-3^+^CD8^+^ T cells and PD-1^+^CD8^+^ T cells were detected in the TG.

Altogether, these findings suggest that similar to HSV-1 infected SYMP humans: (*i*) HSV-specific CD8^+^ T cells in infected cornea and TG of mice show elevated expression of LAG-3 and PD-1 exhaustion markers; and (*ii*) a conspicuous involvement of the LAG-3 and PD-1 pathways in mediating CD8^+^ T cell exhaustion during the latent phase of symptomatic herpes infection.

### Blockade of LAG-3 and PD-1 During Acute Phase Controls Herpes Infection and Disease and Strengthens the Anti-viral Immune Response

We next studied the effect of blocking LAG-3 and PD-1 using antagonist mAbs, on viral infection, disease, and anti-viral CD8^+^ T cell response during the acute phase of HSV-1 infection. A group of 40 WT B6 mice were infected with 2 × 10^5^ pfu of HSV-1 strain McKrae. Mice were intraperitoneally (i.p.) injected with 200 μg of anti-LAG-3 mAb (*n* = 10) and 200 μg of anti PD-1 mAb (*n* = 10) at 3 different time points [i.e., days 3, 5, and 7 post-infection (PI)].

**Figure 3:**
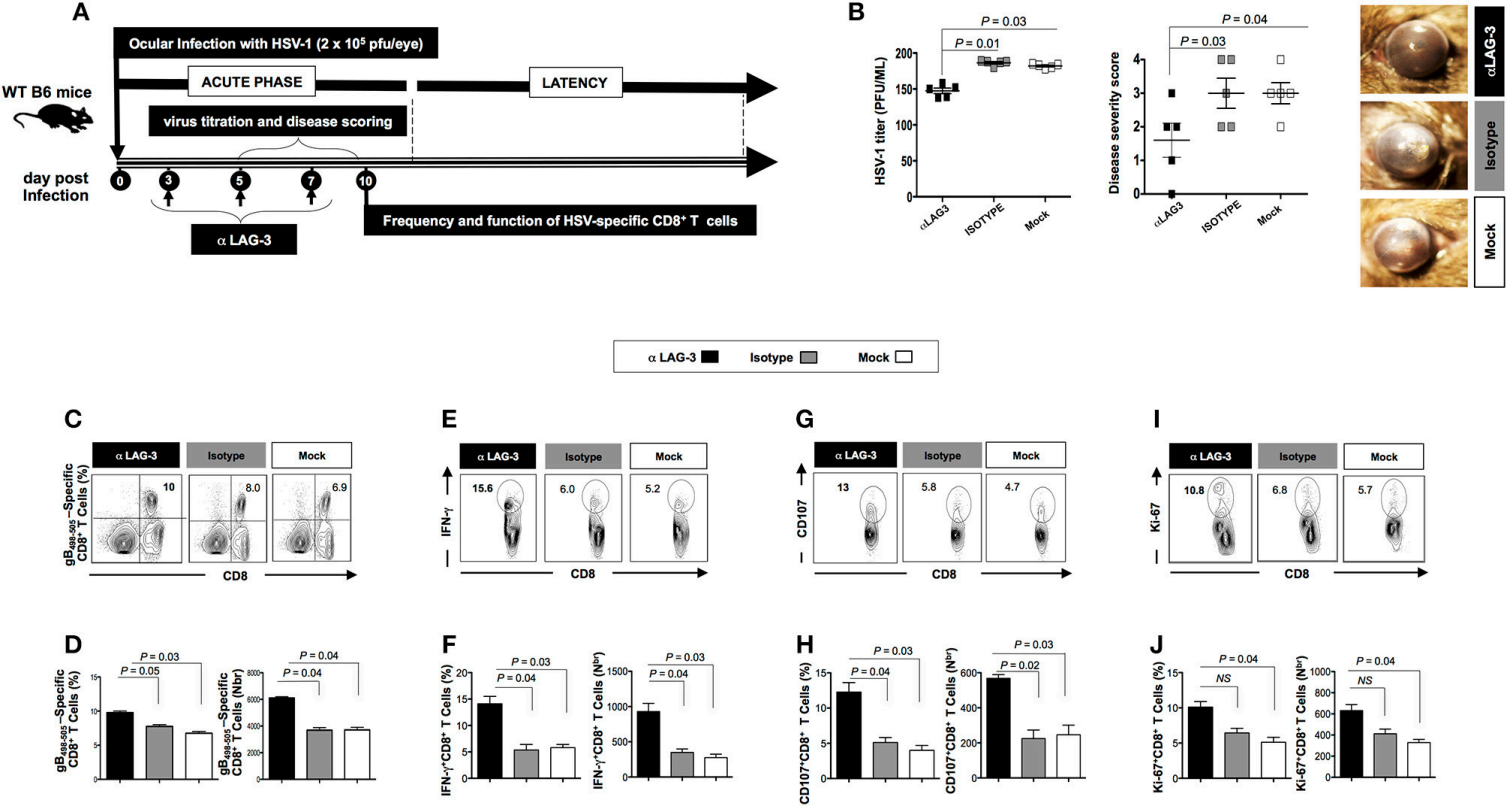
Effects of the LAG-3 blockade on herpes infection, disease and antiviral CD8+ T cell response during the acute phase of ocular herpes infection. **(A)** Schematic representation of HSV-1 ocular infection, blockade LAG-3 mAb treatment, virological and immunological analyses in 30 WT B6 mice (6–8 weeks old) following ocular infection on day 0 with 2 × 10^5^ pfu of HSV-1 (strain McKrae) as detailed in the *Materials* and *Methods* section. **(B)** Quantification of infectious virus particles in the eye swabs by standard plaque assay on RS cells and acute eye disease scoring on a scale of 1–4. Mean viral load (pfu/ml), mean disease score and representative eye disease pictures after the terminal day of the blockade (day 8) are shown. Representative FACS plots, average percentage and average absolute number of **(C,D)** HSV- specific (gB_498−505_) CD8^+^ T cells **(E,F)** HSV-specific IFNy^+^CD8^+^ T cells **(G,H)** HSV-specific CD107^+^CD8^+^ T cells **(I,J)** HSV-specific Ki-67^+^CD8^+^ T cells in the spleen of mice from each group. The results are representative of two independent experiments. The indicated *P*-values, calculated using the unpaired *t*-test, show statistical significance between mAb treated and control groups.

**Figure 4:**
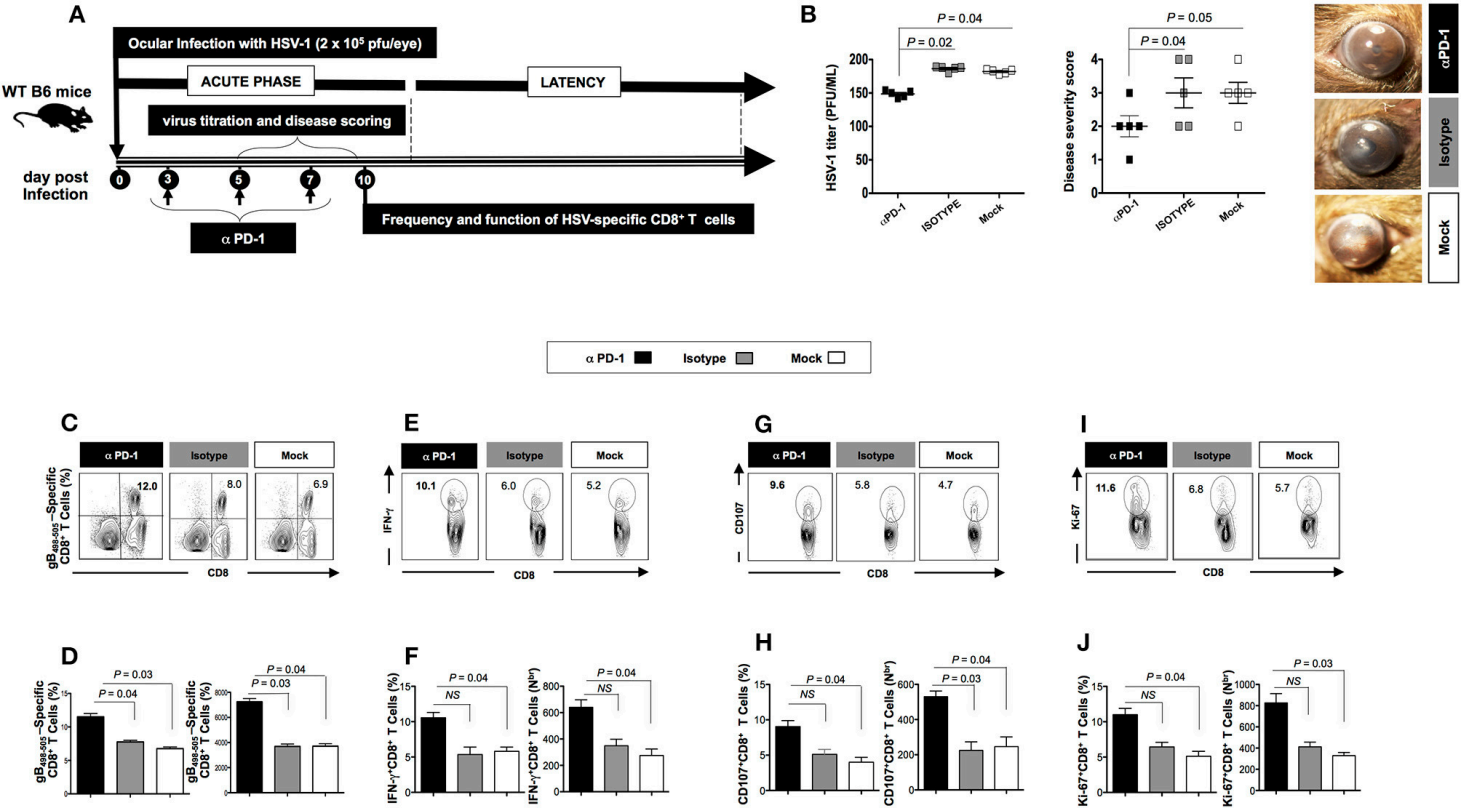
Effect of the PD-1 blockade on herpes infection, disease and antiviral immune response during the acute phase of ocular herpes virus. **(A)** Schematic representation of HSV-1 ocular infection, blockade PD-1 mAb treatment, virological and immunological analyses in 30 WT B6 mice (6–8 weeks old) following ocular infection on day 0 with 2 × 10^5^ pfu of HSV-1 (strain McKrae) as detailed in the *Materials* and *Methods* section **(B)** Quantification of infectious virus particles in the eye swabs by standard plaque assay on RS cells and acute eye disease scored on a scale of 1–4. Mean viral load (pfu/ml), mean disease score and representative eye disease pictures after the terminal day of the blockade (day 8) are shown. Representative FACS plots, average percentage and average absolute number of **(C,D)** HSV- specific (gB_498−505_) CD8^+^ T cells **(E,F)** HSV- specific IFNy^+^CD8^+^ T cells **(G,H)** HSV-specific CD107^+^CD8^+^ T cells **(I,J)** HSV-specific Ki-67^+^CD8^+^ T cells in the spleen of mice from each group. The results are representative of 2 independent experiments. The indicated *P*-values, calculated using the unpaired *t*-test, show statistical significance between mAb treated and control groups.

Following the blockade of both LAG-3 and PD-1, a significant decrease in viral replication and disease was evidenced (*P* < 0.05, Figures 3A,B, 4A,B). This was associated with a significant decrease in the severity of primary ocular disease, as shown in average of 10 mice (**Figures 3B, 4B**, *right panels*) and in representative eye disease pictures (**Figures 3B, 4B**, *left panels*). Further the number and function of HSV-1 gB_495−505_ specific CD8^+^ T cells detected from αLAG-3 (Figures 3C–**J)** and αPD-1(Figures 4C–J) mAb treated mice revealed an increase in both the frequency and number of gB_498−505_ specific CD8^+^ T cells, IFN-γ^+^CD8^+^ T cells, CD107^+^CD8^+^ T cells, and Ki-67^+^CD8^+^ T cells, as compared to isotype and mock treated control groups. Representative FACS plots showed heightened frequencies of gB_498−505_ specific CD8^+^ T cells (αLAG-3: 10% vs. Isotype: 8%, Mock: 6.9%; αPD-1: 12% vs. Isotype: 8%, Mock: 6.9%), IFN-γ^+^CD8^+^ T cells (αLAG-3: 15.6% vs. Isotype: 6%, Mock: 5.2%; αPD-1: 10.1% vs. Isotype: 6%, Mock: 5.2%), CD107^+^CD8^+^ T cells (αLAG-3: 13% vs. Isotype: 5.8%, Mock: 4.7%; αPD-1: 9.6% vs. Isotype: 6%, Mock: 5.2%) and Ki-67^+^CD8^+^ T cells (αLAG-3: 10.8% vs. Isotype: 6.8%, Mock: 5.7%; αPD-1: 11.6% vs. Isotype: 6%, Mock: 5.2%) in αLAG-3 mAb (Figures 3C–**J)** and αPD-1 mAb (Figures 4C–**J**) treated groups in comparison to controls. The corresponding average percentage and average absolute number of gB_498−505_ tetramer specific CD8^+^ T cells; IFN-γ^+^ CD8^+^ T cells; CD107^+^CD8^+^ T cells and Ki-67^+^CD8^+^ T cells are also shown for αLAG-3 (Figures 3C–J) and αPD-1 (Figures 4C–**J**) treated groups in comparison to controls. No systemic or local side effect was detected following the blockade of both LAG-3 and PD-1 pathways.

Altogether, these results suggest that blockade of the LAG-3 and PD-1 pathways of exhaustion can be a promising strategy to combat ocular herpes. As PD-1 blockade is already widely reported to combat persistent pathogens including HSV and is the most thoroughly investigated immune checkpoint pathways, our results essentially reinforce the earlier studies, and henceforth we focused on the lesser investigated LAG-3 pathway to combat HSV reactivation.

### Combination of a Therapeutic LAG-3 Blockade and Therapeutic Immunization Restored the Function of HSV-Specific CD8^+^ T Cells in Cornea and TG Associated With a Reduction in Recurrent Ocular Herpes

**Figure 5:**
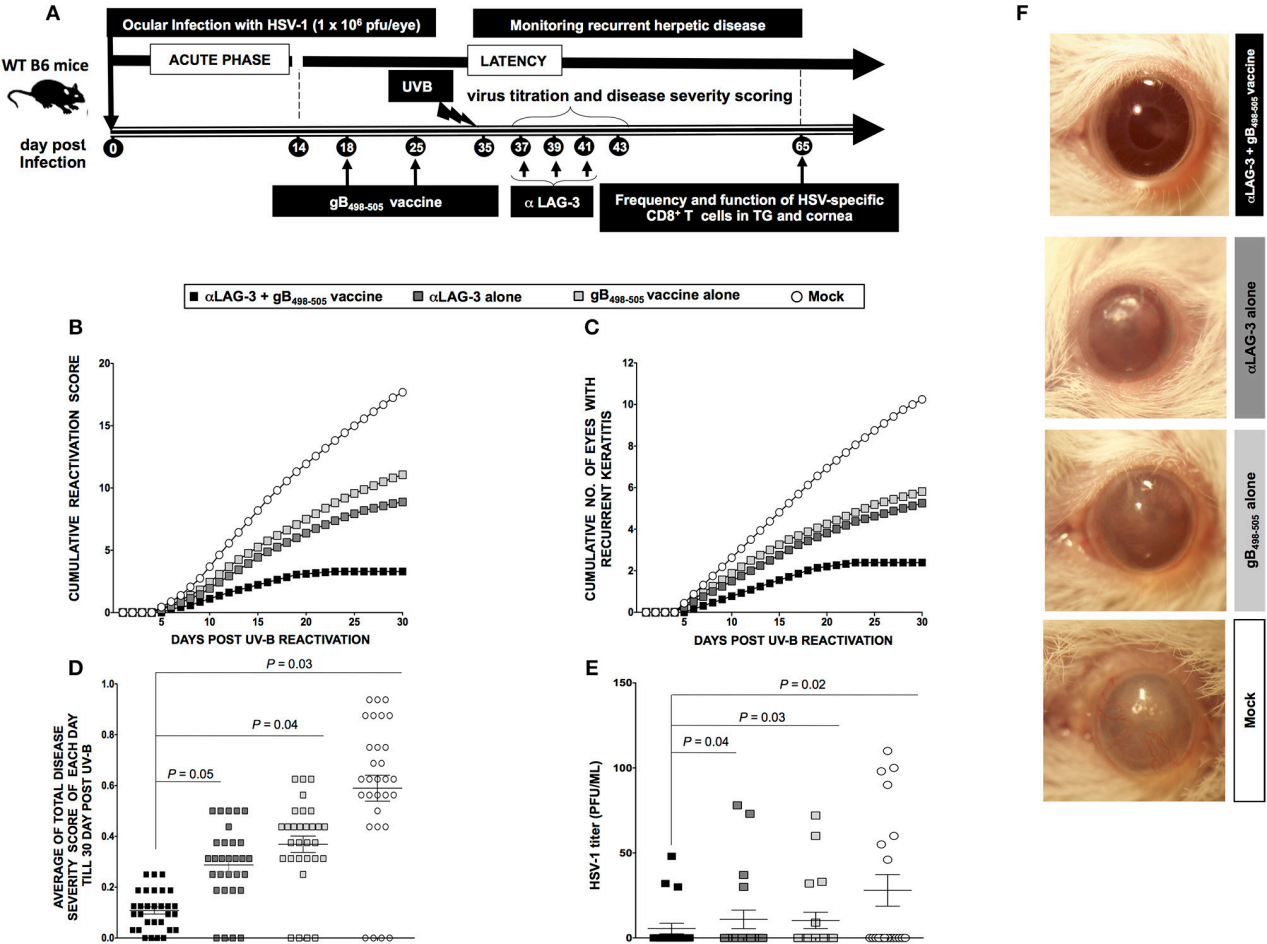
Blockade of LAG-3 following UV-B induced HSV-1 reactivation reduces recurrent ocular herpes. **(A)** Schematic representation of UV-B induced reactivation, blockade by mAb treatment, virological and immunological analyses, and recurrent eye disease scoring in 40 B6(Cg)-*Tyr*^*c*−2*J*^/J mice (6–8 weeks old) categorized into α-LAG-3 + gB_498−505_, α-LAG-3 alone, gB_498−505_ alone, and mock groups on the basis of different immunization strategies following ocular infection on day 0 with 1 × 10^6^ pfu of HSV-1 (strain McKrae). **(B)** Cumulative reactivation score till 30-day post UV-B. **(C)** The cumulative number of eyes showing recurrent disease till 30-day post UV-B. **(D)** Average of total eye disease score of each day till 30-day post UV-B. **(E)** Average viral shedding evaluation by plaque assay after the last day of LAG-3mAb treatment (day 8 post UV-B). **(F)** Representative eye pictures of recurrent disease. Results are representative of two independent experiments. The indicated *P*-values, calculated using the unpaired *t*-test, show statistical significance between α-LAG-3 + gB_498−505_ and other groups.

Subsequently, we determined whether LAG-3 blockade after HSV-1 reactivation from latently infected mice would reduce viral shedding and disease and restore antiviral immune response. We used our novel UV-B model of virus reactivation and preferred albino B6(Cg)-*Tyr*^*c*−2*J*^/J mice over WT B6 mice, as they are known to be more susceptible to reactivation and recurrent corneal disease. Four groups of B6(Cg)-*Tyr*^*c*−2*J*^/J mice (*n* = 10/group) were latently infected with 1 × 10^6^ pfu of McKrae as described in the *Materials* and *Methods* and were then segregated as follows: (1) a group of αLAG-3 + gB_498−505_ mice was therapeutically immunized during latency on days 18 and 25 PI with gB_498−505_ CD8^+^ T cell epitope mixed with the CD4^+^ T helper epitope PADRE and then treated three times with αLAG-3 on days 3, 5, and 7 post UV-B exposure (i.e., days 37, 39, and 41 PI, respectively); (2) a group of αLAG-3 alone, in which mice were non-immunized but therapeutically treated with αLAG-3 as in 1; (3) a group of gB_498−505_ alone, in which mice were only immunized with gB_498−505_ CD8^+^ T cell epitope mixed with the CD4^+^ T helper epitope PADRE; and (4) a group of Mock controls, in which the mice were neither immunized nor treated with αLAG-3. All the three forms of therapeutic interventions: αLAG-3 + gB_498−505_/PADRE, αLAG-3 alone, gB_498−505_/PADRE alone significantly reduced the manifestation of UV-B induced recurrent disease compared to mock, but the combination therapy of αLAG-3 + gB_498−505_/PADRE showed the most significant effect (Figures 5A–D). This is clear from both the cumulative reactivation score (Figure 5B) and the cumulative number of eyes with recurrent disease (Figure 5C). The average score of each day per group detected till day 30 post UV-B exposure also revealed significant difference between the combination therapy of αLAG-3 + gB_498−505_ vaccine vs. αLAG-3 alone (*P* = 0.05), αLAG-3+gB_498−505_ vaccine vs. gB_498−505_ vaccine alone (*P* = 0.04), and αLAG-3+gB_498−505_ vaccine vs. mock (*P* = 0.03) (Figure 5D). Meanwhile, the average degree of viral shedding following the final αLAG-3 treatment (day 8 post UV-B) showed significant difference between combination therapy of αLAG-3 + gB_498−505_ vs. αLAG-3 alone (*P* = 0.04), αLAG-3+gB_498−505_ vs. gB_498−505_ alone (*P* = 0.03), and αLAG-3 + gB_498−505_ vs. mock (*P* = 0.02) (Figure 5E). Representative eye pictures showed significant differences in disease severity amongst all the groups (Figure 5F).

**Figure 6:**
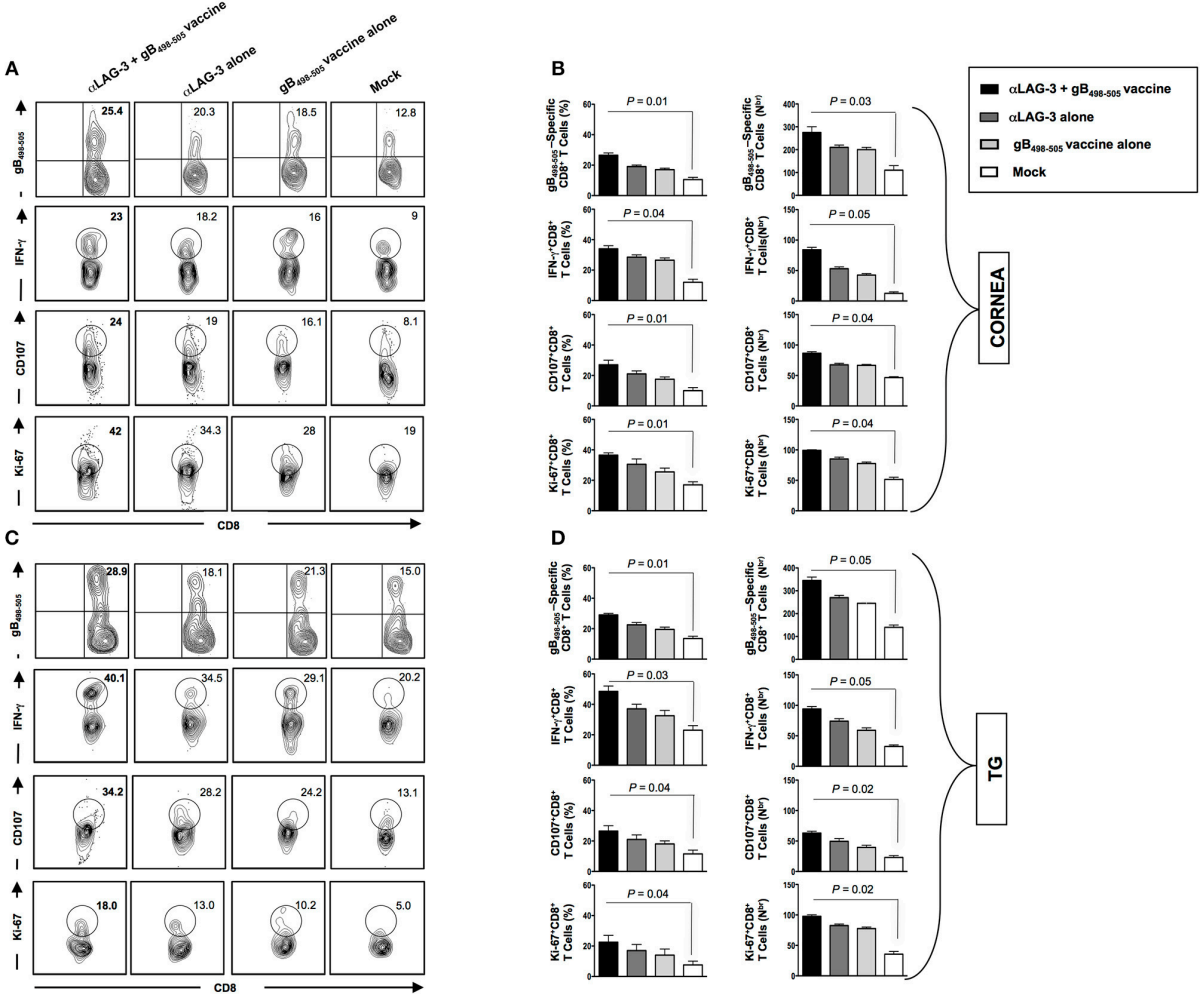
Reduction in recurrent disease by blockade of LAG-3 is associated with better anti-viral immune response in cornea and TG. Representative FACS plots, average percentage and average absolute number of HSV- specific (gB_498−505_) CD8^+^ T cells; HSV- specific IFNy^+^CD8^+^ T; HSV-specific CD107^+^CD8^+^ T cells; HSV-specific Ki-67^+^CD8^+^ T cells against four immunization strategies namely α-LAG-3 +gB_498−505_ vaccine, α-LAG-3 alone, gB_498−505_ vaccine alone, and mock in **A,B** cornea and **(C,D)** TG are shown. Results are representative of 2 independent experiments. The indicated *P*-values are calculated using the unpaired *t*-test, show statistical significance between α-LAG-3 + gB_498−505_ vaccine and mock groups.

At the end of monitoring recurrent disease on day 30 post UV-B, we sacrificed the mice of all groups, harvested mononuclear cells (MNC's) from cornea and TG, as described in *Materials* and *Methods* and determined the number and function of HSV-specific CD8^+^ T cells. Representative FACS plots showed increased frequencies of gB_498−505_ specific CD8^+^ T cells (Cornea: αLAG-3 + gB_498−505_ vaccine: 25.4% vs. αLAG-3 alone: 20.3%, gB_498−505_ vaccine alone: 18.5%, Mock: 12.8%; TG: αLAG-3+gB_498−505_ vaccine: 28.9% vs. αLAG-3 alone: 18.1%, gB_498−505_ vaccine alone: 21.3%, Mock: 15%), IFN-γ^+^CD8^+^ T cells (Cornea: αLAG-3 + gB_498−505_ vaccine: 23% vs. αLAG-3 alone: 18.2%, gB_498−505_ vaccine alone: 16%, Mock: 9%; TG: αLAG-3 + gB_498−505_ vaccine: 40.1% vs. αLAG-3 alone: 34.5%, gB_498−505_ vaccine alone: 29.1%, Mock: 20.2%), CD107^+^CD8^+^ T cells (Cornea: αLAG-3 + gB_498−505_ vaccine: 24% vs. αLAG-3 alone: 19%, gB_498−505_ vaccine alone: 16.1%, Mock: 8.1%; TG: αLAG-3 + gB_498−505_ vaccine: 34.2% vs. αLAG-3 alone: 28.2%, gB_498−505_ vaccine alone: 24.2%, Mock: 13.1%), and Ki-67^+^CD8^+^ T cells (Cornea: αLAG-3 + gB_498−505_ vaccine: 42% vs. αLAG-3 alone: 34.3%, gB_498−505_ vaccine alone: 28%, Mock: 19%; TG: αLAG-3 + gB_498−505_ vaccine: 18% vs. αLAG-3 alone: 13%, gB_498−505_ vaccine alone: 10.2%, Mock: 5%) in combination therapy of αLAG-3 + gB_498−505_ vaccine group when compared with the other groups (Figures 6A,C). The corresponding average percentage and average absolute number of gB_498−505_ tetramer specific CD8^+^ T cells; IFN-γ^+^ CD8^+^ T cells; CD107^+^CD8^+^ T cells and Ki-67^+^CD8^+^ T cells are also shown for all the groups (αLAG-3+gB_498−505_ vaccine, αLAG-3 alone, gB_498−505_ vaccine alone, and mock) in cornea (Figure 6B) and TG (Figure 6D).

Taken together, these results suggest that the combined effect of the blockade and immunization (1) significantly combats the manifestation of disease severity during reactivation of HSV-1 from latency and (2) significantly restores HSV specific immunity in the resident tissues during latency that underscores the observed protection from recurrent ocular herpes.

### Therapeutic Immunization Improves HSV-Specific CD8^+^ T Cell Response in the Cornea and TG and Protects Against Recurrent Ocular Herpes in LAG-3^−/−^ Mice

**Figure 7:**
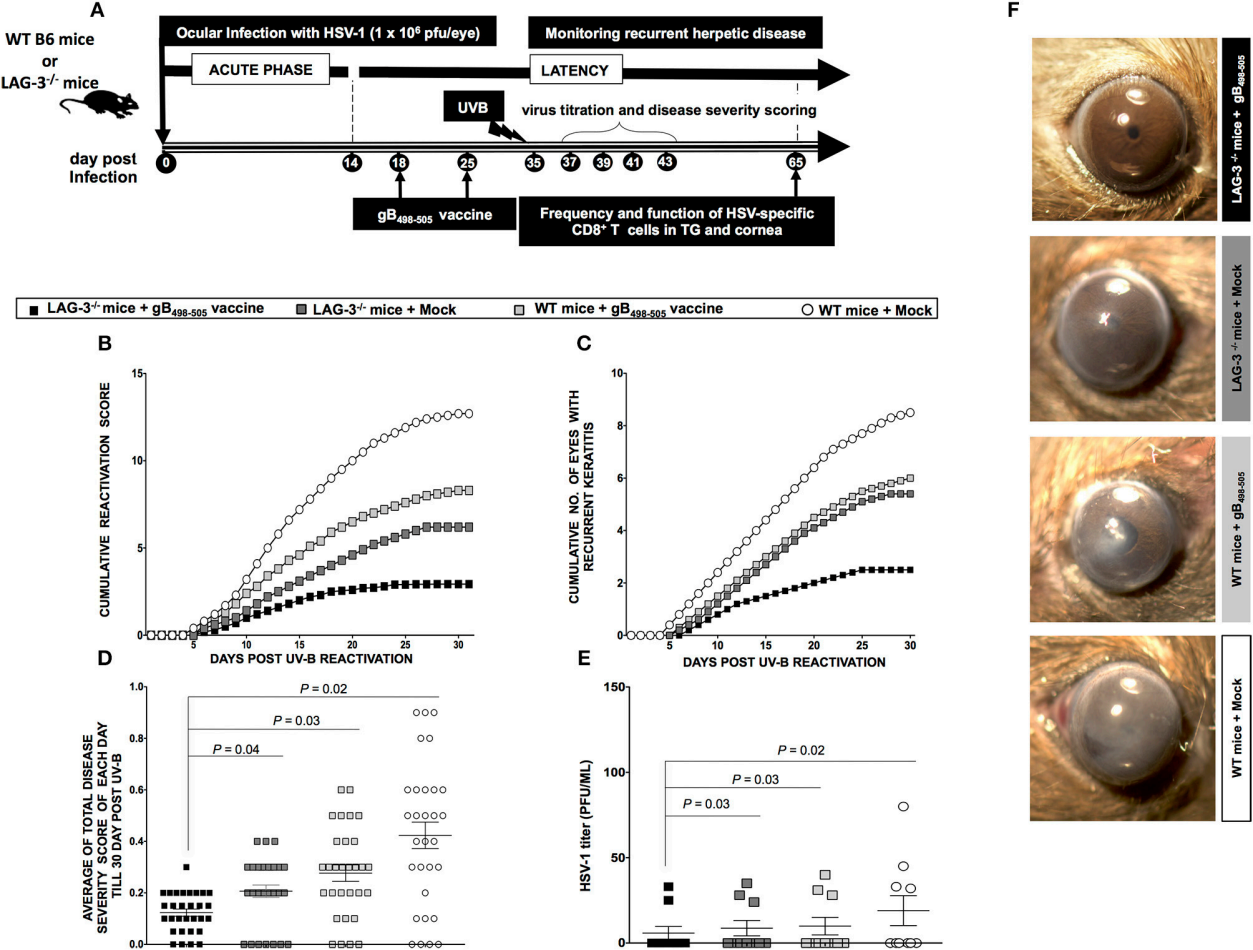
LAG-3^−/−^ mice show less recurrent ocular herpes compared to WT B6 mice following UV-B induced HSV-1 reactivation. **(A)** Schematic representation of UV-B induced reactivation, virological and immunological analyses, following HSV-1 ocular infection of 20 WT B6 and 20 LAG-3^−/−^ mice (6–8 weeks old) on day 0 with 1 × 10^6^ pfu of HSV-1 (strain McKrae). In one group; WTs B6 mice (*n* = 10) and LAG-3^−/−^ mice (*n* = 10) are immunized; while in the other group, WTs B6 (*n* = 10) and LAG-3^−/−^ (*n* = 10) mice remain non-immunized. **(B)** The cumulative reactivation score till day 30 post UV-B. **(C)** The cumulative number of eyes showing recurrent disease till day 30 post UV-B. **(D)** Average of total eye disease score of each day till day 30 post UV-B. **(E)** Average viral shedding measured by plaque assay at day 8 post UV-B. **(F)** Representative eye pictures of recurrent disease. Results are representative of 2 independent experiments. The indicated *P*-values, calculated using the unpaired *t*-test, show statistical significance between LAG-3^−/−^ + gB_498−505_ and other groups.

Two groups of mice (WT B6 and LAG-3^−/−^ deficient mice (*n* = 10/group) were latently infected with 1 × 10^6^ pfu of McKrae. One group of WT B6 and one of LAG-3^−/−^ mice were immunized with the immunodominant gB_498−505_ peptide mixed with the promiscuous CD4^+^Th epitope PADRE and CpG_1826_ adjuvant as detailed in the *Materials* and *Methods* section (Figure 7A). The groups were then divided as follows: (1) LAG-3^−/−^ mice + gB_498−505_ vaccine; (**2**) LAG-3^−/−^ mice + Mock vaccine; (3) WT mice + gB_498−505_ vaccine; and (4) WT mice + Mock vaccine.

As shown in Figures 7B–**D**, the groups LAG-3^−/−^ + gB_498−505_ vaccine, LAG-3^−/−^ + Mock vaccine, WT + gB_498−505_ vaccine showed significant reduction of recurrent disease compared to WT + Mock vaccine. Moreover, the group of mice that received the combination therapy of the LAG-3^−/−^ + gB_498−505_ vaccine showed the most significant effect on recurrent corneal herpetic disease. This is evident from both the cumulative reactivation score (Figure 7B) and the cumulative number of eyes with recurrent disease (Figure 7C). As shown in Figure 7D the average of total score of each day per group, detected up to 30-day post UV-B induced reactivation, showed a significant difference between; LAG-3^−/−^ + gB_498−505_ vaccine vs. LAG-3^−/−^ + Mock (*P* = 0.04), LAG-3^−/−^ + gB_498−505_ vaccine vs. WT + gB_498−505_ vaccine (*P* = 0.03), LAG-3^−/−^ + gB_498−505_ vaccine vs. WT + Mock (*P* = 0.02).

Significance differences in virus shedding were found between LAG-3^−/−^ + gB_498−505_ vaccine vs. LAG-3^−/−^ + Mock (*P* = 0.03), LAG-3^−/−^ + gB_498−505_ vaccine vs. WT + gB_498−505_ vaccine (*P* = 0.03), LAG-3^−/−^ + gB_498−505_ vaccine vs. WT + Mock (*P* = 0.02) (Figure 7E). Recurrent disease was also significantly reduced in LAG-3^−/−^ + gB_498−505_ vs. all the other groups (Figure 7F).

**Figure 8:**
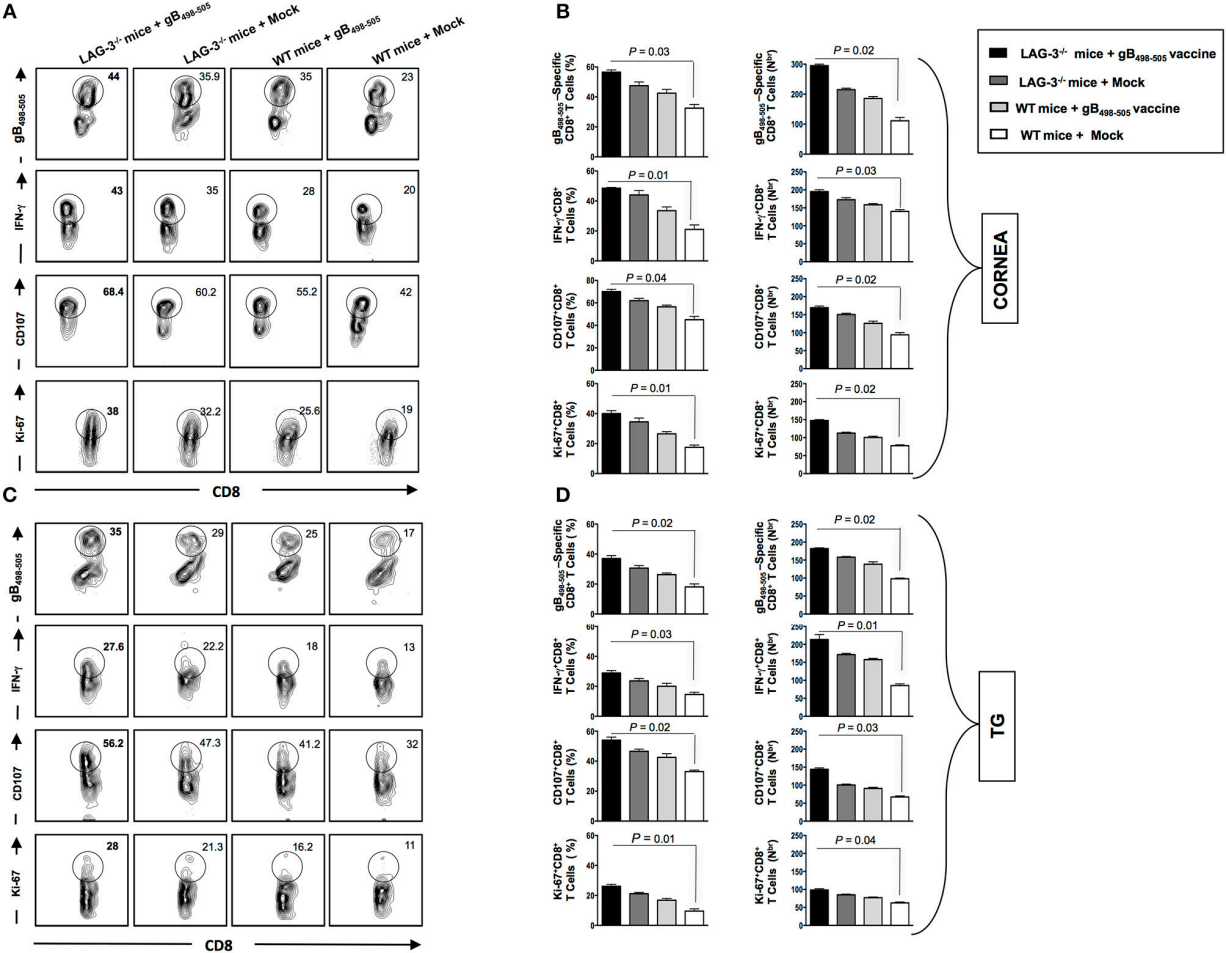
Reduction in recurrent disease in LAG-3^−/−^ mice is associated with better anti-viral immune response in cornea and TG. The mice are monitored till day 30 post UV-B and subsequently sacrificed to process cornea, and TG for immunological analysis as mentioned in the *Materials and Methods*. Representative FACS plots, average percentage and average absolute number of HSV- specific (gB_498−505_) CD8^+^ T cells; HSV- specific IFNy^+^CD8^+^ T; HSV-specific CD107^+^CD8^+^ T cells; HSV-specific Ki-67^+^CD8^+^ T cells detected in **A,B** cornea and **(C,D)** TG are shown. The results are representative of 2 independent experiments. The indicated *P*-values, calculated using an unpaired *t*-test, show statistical significance between LAG-3^−/−^ + gB_498−505_ vaccine and mock groups.

On day 30 post UV-B exposure (i.e., at the end of monitoring recurrent disease) the mice of all groups were sacrificed, mononuclear cells (MNC's) from cornea and TG were harvested and antiviral CD8^+^ T cell responses were compared between groups. From the representative FACS plots heightened frequencies for gB_498−505_ specific CD8^+^ T cells (Cornea: LAG-3^−/−^ + gB_498−505_ vaccine: 44% vs. LAG-3^−/−^ + Mock: 35.9%, WT + gB_498−505_ vaccine: 35%, WT + Mock: 23%; TG: LAG-3^−/−^ + gB_498−505_ vaccine: 35% vs. LAG-3^−/−^ + Mock: 29%, WT + gB_498−505_ vaccine: 25%, WT + Mock: 17%), IFN-γ^+^CD8^+^ T cells (Cornea: LAG-3^−/−^ + gB_498−505_ vaccine: 43% vs. LAG-3^−/−^ + Mock: 35%, WT + gB_498−505_ vaccine: 28%, WT + Mock: 20%; TG: LAG-3^−/−^ + gB_498−505_ vaccine: 27.6% vs. LAG-3^−/−^ + Mock: 22.2%, WT + gB_498−505_ vaccine: 18%, WT + Mock: 13%), CD107^+^CD8^+^ T cells (Cornea: LAG-3^−/−^ + gB_498−505_ vaccine: 68.4% vs. LAG-3^−/−^ + Mock: 60.2%, WT + gB_498−505_ vaccine: 55.2%, WT + Mock: 42%; TG: LAG-3^−/−^ + gB_498−505_ vaccine: 56.2% vs. LAG-3^−/−^ + Mock: 47.3%, WT + gB_498−505_ vaccine: 41.2%, WT + Mock: 32%), and Ki-67^+^CD8^+^ T cells (Cornea: LAG-3^−/−^ + gB_498−505_ vaccine: 38% vs. LAG-3^−/−^ + Mock: 32.2%, WT + gB_498−505_ vaccine: 25.6%, WT + Mock: 19%; TG: LAG-3^−/−^ + gB_498−505_ vaccine: 28% vs. LAG-3^−/−^ + Mock: 21.3%, WT + gB_498−505_ vaccine: 16.2%, WT + Mock: 11%) were observed in combination therapy of LAG-3^−/−^ + gB_498−505_ vaccine group when compared with the other groups (Figures 8A,C). The corresponding average percentage and average absolute number of gB_498−505_ tetramer specific CD8^+^ T cells; IFN-γ^+^ CD8^+^ T cells; CD107^+^CD8^+^ T cells and Ki-67^+^CD8^+^ T cells are also shown for all the groups (LAG-3^−/−^ mice + gB_498−505_, LAG-3^−/−^ mice + Mock, WT mice + gB_498−505_, WT mice + Mock) in cornea (Figure 8B) and TG (Figure 8D). Taken together, these results essentially substantiate our earlier observations on the blockade of LAG-3.

## Discussion

An immune-surveillance role for the trigeminal ganglia-resident HSV-specific CD8^+^ T cells that decrease virus reactivations has been established (9, 10, 28, 36–38). However, the mechanisms of CD8^+^ T cell dynamics in recurrent ocular herpetic disease remain to be fully elucidated. Key knowledge gaps that remain include: (*i*) How CD8^+^ T cells protect from reactivation, virus shedding, and recurrent disease; and (*ii*) The immune evasion strategies evolved by the virus as a counter-defense against the host's CD8+ T cells. How does the virus evade CD8^+^ T cell immune surveillance to allow efficient reactivation from latency? This report shows that: (*i*) Humans with recurrent clinical ocular herpes upregulate expression of the inhibitory receptors PD-1 and LAG-3 on their HSV-specific CD8^+^ T cells; (*ii*) Compared to ASYMP individuals, SYMP individuals have a significant number of PD-1^+^CD8^+^ T cells and LAG-3^+^CD8^+^ T cells; (*iii*) HSV-1 specific CD8^+^ T cells from SYMP patients, but not from healthy ASYMP individuals, were functionally exhausted; (*iv*) In mice latently infected with HSV-1(strain McKrae), there is an increase in cornea- and TG-resident LAG-3^+^CD8^+^ and PD-1^+^CD8^+^ T cells. LAG-3^+^CD8^+^ T and PD-1^+^CD8^+^ T cells increased in both TG and cornea as early as 3 days of acute infection, and on day 14-post-infection (just after the acute infection has cleared), there were significantly more LAG-3^+^CD8^+^ T cells and PD-1^+^CD8^+^ T cells in both the cornea and TG. However, on days 23 to 41, during latent infection, the number of both LAG-3^+^CD8^+^ T cells and PD-1^+^CD8^+^ T cells started to decline. To our knowledge, this is the first report showing: (*i*) LAG-3-related functional exhaustion of HSV-1 specific CD8^+^ T cells in both TG and cornea of latently infected mice; (*ii*) A significant increase in functionally exhausted LAG-3^+^CD8^+^ T cells in SYMP patients as well as in B6 mice that developed increased virus shedding and severe recurrent corneal herpetic disease following UV-B induced reactivation.

This study also confirms our previous finding showing that HSV-1 infection in B6 mice results in accumulation of virus-specific exhaustion CD8^+^T cells, expressing PD-1, in latently-infected trigeminal ganglia (TG) (10, 15, 28). More importantly, we show that combination of a therapeutic LAG-3 blockade and therapeutic immunization restored the function of HSV-specific CD8^+^ T cells in cornea and TG associated with a reduction in recurrent ocular herpes, following UV-B induced reactivation in latently infected B6 mice. Thus, in addition to previously described immune evasion mechanisms (5, 8, 15, 28, 29, 29, 39–44), our data reveal a novel mechanism by which HSV-1 evades the protective host immune responses through dampening and dysregulating LAG-3^+^CD8^+^ T cell function. Moreover, the LAG-3 pathway plays a fundamental role in ocular herpes T cell immunity, thus providing an important immune checkpoint target that can be combined with T cell-based therapeutic vaccines to improve protection against recurrent ocular herpes.

A protective CD8^+^ T cell response to viral infection depends upon T cell receptor (TCR) stimulation along with costimulatory signals (45). Dysregulation in positive and negative co-stimulatory signals affects the magnitude of CD8^+^ T cell response (45–47). The LAG-3 receptor is a negative T cell co-stimulatory molecule that is highly expressed on dysfunctional virus specific CD8^+^ T cells and interacts with MHC-II (48). In this report, we found that LAG-3 is highly expressed on HSV-specific CD8^+^ T cells from symptomatic (SYMP) individuals, in which HSV-1 reactivation often causes painful recurrent corneal disease (49–52). In contrast, LAG-3 was comparatively low on HSV-specific CD8^+^ T cells from asymptomatic (ASYMP) individuals in which virus reactivation never causes recurrent disease (31, 34, 52, 53). Our results in humans suggest that the magnitude of the HSV-specific CD8^+^ T cell immunity, after ocular HSV-1 reactivation, is subject to control by the LAG-3 pathway.

The high level of LAG-3 expression were detected on HSV-1 VP11/12_66−74_ epitope-specific human CD8^+^ T cells as well on CD8^+^ T cells specific to four additional HSV-1 epitopes from VP13/14, gB, UL43, and UL44 proteins (Coulon et al., Manuscript in preparation). However, no upregulation was detected on bulk CD8^+^ T cells, suggesting that the observed T cell exhaustion is restricted to herpes-specific LAG3^+^CD8^+^ T cells. The ultimate underlying molecular mechanism by which LAG-3 pathway led to CD8^+^ T cell exhaustion is not defined by the present study. However, as illustrated in Figure S2, the findings are consistent with a potential role of LAG-3 pathway in exhaustion of HSV-specific CD8^+^ T cells and that mAbs blocking of such pathways reverse such dysfunction associated with protection from recurrent ocular herpes. MHC class II is the main ligand of LAG-3 receptor (54). MHC-II appears to bind with higher affinity to LAG-3 compared to CD4 molecule a, competition that is expected to destabilized the TCR/CD4/MHC-II interaction (55, 56). Doing so, the LAG-3 pathway negatively regulates the function and homeostasis of CD4^+^ T cells (57, 58). Moreover, LAG-3 pathway also regulates the function and homeostasis of CD8^+^ T cells during chronic viral infection (48). The mechanisms by which LAG-3 pathway regulated CD8^+^ T cells function/dysfunction remain to be fully elucidated. It is also unclear whether LAG-3 competition with MHC class II makes HSV-specific CD4^+^ T cells more exhausted than HSV-specific CD8^+^ T cells, mainly because of lack of CD4^+^ T cell specific tetramers that would help quantify the function HSV-specific CD4^+^ T cells. Nevertheless, we found that regulation of CD8^+^ T cell exhaustion by LAG-3 and PD-1 inhibitory pathways was non-redundant, as blockade of the T cell inhibitory receptors LAG-3 and PD-1 simultaneously and synergistically improved CD8^+^ T cell responses and diminished HSV-1 load and recurrent disease in HLA Tg mice (Roy et al., under review). Thus, antiviral CD8^+^ T cell responses during herpes viral infection appears to be regulated by complex patterns of co-expressed inhibitory receptors.

During HSV-1 neuronal latency in the TG of mice and humans, a small number of latently infected neurons are surrounded by CD8^+^ T cells (9, 59–61). Since CD8^+^ T cells are presumably attracted to these neurons by viral antigens, it is assumed that the neurons surrounded by CD8^+^ T cells are those in which the virus has initiated the early stages of reactivation from latency. In mice, experimental reactivation of HSV-1 from latency is typically accomplished by explanting TG into tissue culture media for up to 14 days and testing for the appearance of infectious (i.e., reactivated) virus (29, 29, 30). In this TG explant induced reactivation model, depleting CD8^+^ T cells with specific mAb leads to the detection of more reactivated virus (9, 62). Conversely, addition of exogenous CD8^+^ T cells reduces detection of reactivated virus (6, 9, 11). Thus, with wild type HSV-1, CD8^+^ T cells in the TG are apparently able to reduce the detection of infectious reactivated virus. Following T cell activation *in vivo*, many co-stimulatory and inhibitory receptors are upregulated on T cells. It is possible that inhibitory interactions between the LAG-3 receptor on T cells and its respective ligand on APCs, such infected DCs and MΦ, at the time of priming are attenuating the effector T cell response. In fact, we found that HSV-1 infection of mice lacking LAG-3 expression on hematopoietic cells (i.e., LAG-3^−/−^ deficient mice) generated more HSV-specific IFN-γ-producing cytotoxic CD107^+^CD8^+^ T cells compared to wild type (WT) infected mice. Moreover, we demonstrated that blocking the LAG-3 *in vivo* following administration of anti-LAG-3-specific mAbs at the time of T cell priming significantly enhanced the number of HSV-1 gB_498−−505_-specific CD8^+^ T cells in resident tissues. We also demonstrated that blocking the LAG-3 pathway at the time of priming increases the frequency of IFN-γ-secreting HSV-1 gB_498−−505_-specific CD8^+^ T cells and improves their cytotoxic potential. It is possible that the inhibitory receptor LAG-3 might be involved in regulating the effector function of HSV specific CD8^+^ T cells.

The recent success of therapies targeting immune checkpoints to treat many cancers has driven a reappearance of interest in therapies targeting immune checkpoints against chronic infections and diseases (63–65). While still in its early stages, basic and clinical data suggest that blockade of CTLA-4 and PD-1 can be beneficial in the treatment of chronic HIV, HBV, and HCV infection, as well as other chronic diseases. Furthermore, novel inhibitory receptors such as TIM-3, LAG-3, and TIGIT are the potential next wave of checkpoints that can be manipulated for the treatment of chronic infections (64). However, caution should be taken when blocking immune checkpoint pathways that help keep the body's immune responses in check. Releasing the “brakes” on the immune system over-activate effector CD8^+^ T cells that might cause tissue damage. Both PD-1 and LAG-3 play important roles during the normal immune response to prevent autoimmunity. Nevertheless, in the present study we found that mAbs therapies blocking LAG-3 immune checkpoint safely and efficiently led to significant reductions of recurrent corneal herpetic disease following UV-B induced reactivation in B6 mice latently infected with HSV-1. The improved clinical outcome of LAG-3 blockade in mice with established UV-B induced recurrent herpes was directly associated with a multifaceted enhancement of both the numbers of function antiviral CD8^+^ T cells. Moreover, this report shows for the first time that a combination of a therapeutic blockade of LAG-3 immune checkpoint and a therapeutic vaccination leads to the generation of functional HSV-specific CD8^+^ T cells in latently infected TG and cornea associated with an even more reduction in virus reactivation and recurrent disease in latently infected mice, following UV-B induced reactivation. No systemic or local side effects were observed following PD-1 and LAG-3 blockade in HSV-1 infected mice pointing to the safety of this treatment. The precise mechanisms by which LAG-3 blockade results in robust numerical and functional enhancement of effector CD8^+^ T cells remain to be discovered. Our data also demonstrate that the “exhausted” phenotype (i.e., PD-1^+^CD8^+^ T cells and LAG-3^+^CD8^+^ T cells) was predominantly established prior to terminal cell differentiation, at the stage of memory-like T cells. It is likely that compartmentalization of inhibitory receptor expression predicts distinct cellular responses to inhibitory receptor blockade. For example, LAG-3 blockade may preferentially act on the terminally differentiated CD8^+^ T cells. On the other hand, PD-1 blockade will act on both the memory-like CD8^+^ T cells and on the terminally differentiated CD8^+^ T cells. Regardless of the mechanisms, since HSV-1 specific CD8^+^ T cells in SYMP individuals appeared to be functionally exhausted with a significant number of PD-1^+^CD8^+^ and LAG-3^+^CD8^+^ T cells, blockade of the LAG-3 and PD-1 signaling transduction pathways provide new therapeutic options for herpes infected symptomatic patients.

The immune checkpoints are often divided into a first and a second-generation (66-70). The classic examples of the first-generation checkpoints are PD-1/PD-L1 and CTLA4 and of the second-generation checkpoints are LAG-3, TIGIT, VISTA, and TIM-3. While blocking of the first-generation molecules is widely employed, a recent shift in focus toward targeting the second-generation molecules is noteworthy, as resistance to first generation therapies are amply reported and a combination of the two show synergistic and non-redundant effects. Some recent reports identify LAG-3, other than the widely studies PD-1, as a powerful inhibitory receptor whose blockade improves T cell immunity and limits diseases (45, 64, 66–68). Blackburn et al. (48) showed that T cell co-expressing multiple inhibitory receptors correlated with a more severe exhaustion and a greater disease load and blockade of LAG-3 and PD-1 show a synergistic effect. LAG-3 is shown to regulate homeostatic proliferation of CD8^+^ T cells and potentiate the suppressor function of regulatory T cells. The KIEELE motif in the cytoplasmic region of LAG-3 has been shown to play a decisive role in its inhibitory effect, although the detailed mechanism is still poorly understood (69, 70).

In this report, we found that the percentage and absolute number of HSV specific IFN-γ^+^CD8^+^T cells, CD107^+^CD8^+^T cells and Ki-67^+^CD8^+^T cells were all significantly decreased in TG and cornea of latently infected mice following UV-B induced reactivation compared to latently infected mice with no induced reactivation (*data not shown*). This indicated phenotypic and functional exhaustion of both cytokine expression and cytotoxic activity of these CD8^+^ T cells. To our knowledge, this is the first report to show significantly more phenotypically and functionally exhausted HSV-specific CD8^+^ T cells in both the TG and cornea of latently infected mice following UV-B induced reactivation. Thus, our original hypotheses that increasing the number of exhausted CD8+ T cells in the TG and cornea led to HSV-1 escape from the control of CD8^+^ T cells was correct.

The increased CD8^+^ T cell exhaustion in TG and cornea of mice infected with HSV-1 could be due to increased viral antigens in these tissues. However, during latency in mouse TGs, less than 1 neuron/TG had detectable viral Ag by immunostaining (8, 71, 72). In addition, if spontaneous reactivation occurs in the mice TG, it is minimal (73). Thus, even though CD8^+^ T cells are much more sensitive to Ag than the antibodies used for immunostaining, the very low un-sustained Ag level that appears to be the situation in the mice TGs is unlikely to result in exhaustion of CD8^+^ T cells. Thus, it seems unlikely that the CD8^+^ T cell exhaustion detected was due to the viral Ag load, unless additional factors contributed to immune stimulation. For example, cornea- and TG-resident HSV-specific CD8^+^ T cells could have a higher functional avidity (ability to respond to low epitope density) than their counterparts in the periphery (18). Alternatively, CD8^+^ T cell exhaustion may suggest that there is a lot more viral Ag present in the TG and cornea of mice latently infected with HSV-1 than has been previously thought. The HSV-gB_498−505_-epitope and B6 mice were chosen to detect HSV-1 specific CD8^+^ T cells in this study because in this mouse strain the majority (over 60%) of CD8^+^ T cells are directed to this single immunodominant epitope (74–76). The phenotypic and functional exhaustion of CD8^+^ T cells specific to other mouse HSV-1 or human CD8^+^ T cell epitopes in humans still remains to be determined. Using our HLA transgenic mouse model (6, 77), we are currently in the process of assessing the exhaustion of CD8^+^ T cells specific to a set of immunodominant and sub-dominant human CD8^+^ T cell HSV-1 epitopes that we have recently identified as being recognized by these animals.

Both SYMP and ASYMP individuals shed the virus in tears as a result of sporadic reactivation, but only the SYMP individuals manifest lifelong recurrences of herpetic disease, usually multiple times a year and often require continuous antiviral therapy (i.e., Acyclovir and derivatives). In this study, we applied blockade during a brief span following the UV-B induced reactivation and as our results suggest, an appropriate strategy to limit recurrent keratitis in SYMP humans, would be to monitor the SYMP patients for reactivation episodes and apply the blockade therapy during those brief phases of recurrences. Prior therapeutic immunization is expected to reinforce the effect of the blockade. Ideally, several rounds of treatment are expected to boost the generation of polyfunctional CD8^+^ T cells in the TG and cornea, improving their versatility. As impaired T cells responses are among the potential causes of symptomatic shedding (3, 78, 79), generation of sturdier polyfunctional T cells in the TG and cornea is expected to restrain or even nullify future harmful reactivation episodes. However, translational hurdles of the study are the safety and timing of the therapy. Another hurdle would be the delivery of mAbs in appropriate amounts to the immunologically recalcitrant sites, TG and cornea. Since our observations indirectly presuppose a significant delivery of mAbs to TG and cornea, it is likely that the timing of delivery is a crucial. In addition, inflammation associated with reactivation episodes increases tissue permittivity. Thus, with optimum dosage through right route of administration at an appropriate time of recurrences will likely make for best delivery of blocking mAbs to the targeted tissues. Such optimal delivery would interfere with virus reactivation from TG and stop or at least reduce recurrent corneal herpetic disease.

In summary, the present study demonstrates, for the first time, that the cornea and TG from HSV-1 infected mice, with UV-B-induced recurrent corneal disease, present more exhausted HSV-specific PD-1^+^CD8^+^ T cells and LAG-3^+^CD8^+^ T cells. Since functional HSV-specific CD8^+^ T cells appear to be important in decreasing reactivation from latency (9), the higher number of functional HSV-specific CD8^+^ T cells was detected following treatment with mAbs that block the LAG-3 pathway associated reduced reactivation and less severe recurrent disease as compared to mock-treated mice. This is also the first study to report that combination of the LAG-3 immune checkpoint blockade together with a therapeutic vaccination leads to generation of functional HSV-specific CD8^+^ T cells in latently infected TG and cornea associated with even more reduction in virus reactivation and recurrent disease. Blockade of the LAG-3 pathway in combination with vaccination may have great therapeutic promise and open up the possibilities of designing novel combination therapies. This includes therapeutic vaccination and the blockade of T cell exhaustion in confronting HSV-1 reactivation and cure of potentially blinding recurrent ocular herpes.

## Ethics Statement

The manuscript, which has not been submitted elsewhere, does contain both human studies and animal studies, which conform to the Guides for IRB and IACUC published by the US National Institute of Health.

## Author Contributions

Conceived and designed the experiments: SR, P-GC, RS, LB. Performed the experiments: SR, P-GC, RS, HV, GK, SW, TY, MF, VL. Analyzed the data: SR, P-GC, RS, LB. Contributed reagents, materials, analysis tools: SR, P-GC, RS, LB. Wrote the paper: SR, P-GC, RS, LB.

### Conflict of Interest Statement

The authors declare that the research was conducted in the absence of any commercial or financial relationships that could be construed as a potential conflict of interest.
