# Supplementary material for: Blockade of LAG-3 Immune Checkpoint Combined With Therapeutic Vaccination Restore the Function of Tissue-Resident Anti-viral CD8+ T Cells and Protect Against Recurrent Ocular Herpes Simplex Infection and Disease
Source: Front Immunol. 2018 Dec 17;9:2922. doi: 10.3389/fimmu.2018.02922 (PMC6304367; doi:10.3389/fimmu.2018.02922)
Supplement: Supplementary file 1 [file Image_1.pdf]

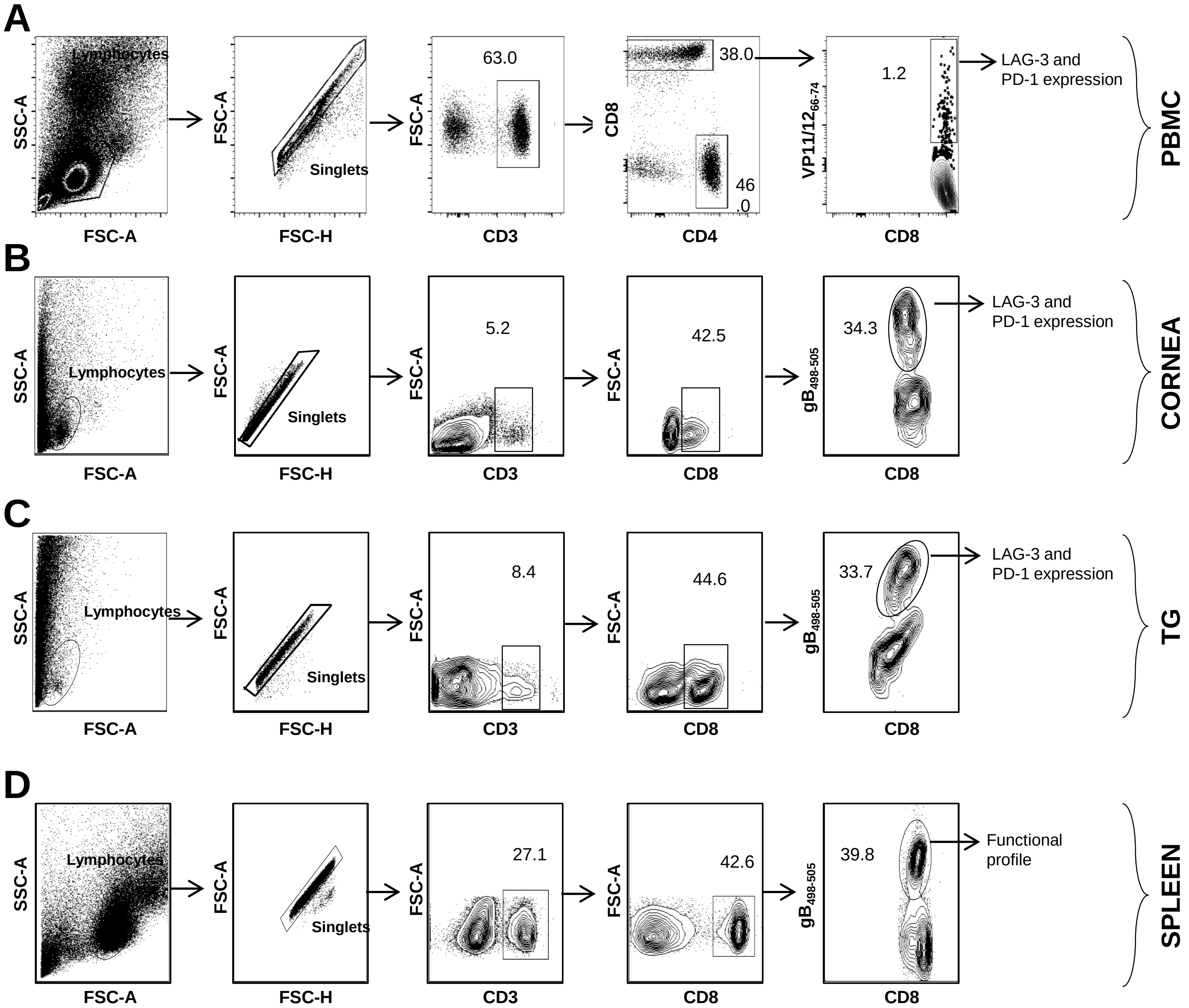

(**A** to **D**) Gating strategies. (**A**) Frequencies of HSV-VP11/12$_{66\text{-}74}$-specific CD8^{+} T cells in humans PBMC. (**B**-**D**) frequencies of mouse HSV-gB$_{498\text{-}505}$ -specific CD8^{+} T cells in different compartments (i.e. CORNEA, TG and SPLEEN).
