# Supplementary figures and images for: Blockade of LAG-3 Immune Checkpoint Combined With Therapeutic Vaccination Restore the Function of Tissue-Resident Anti-viral CD8+ T Cells and Protect Against Recurrent Ocular Herpes Simplex Infection and Disease
Source: Front Immunol. 2018 Dec 17;9:2922. doi: 10.3389/fimmu.2018.02922 (PMC6304367; doi:10.3389/fimmu.2018.02922)

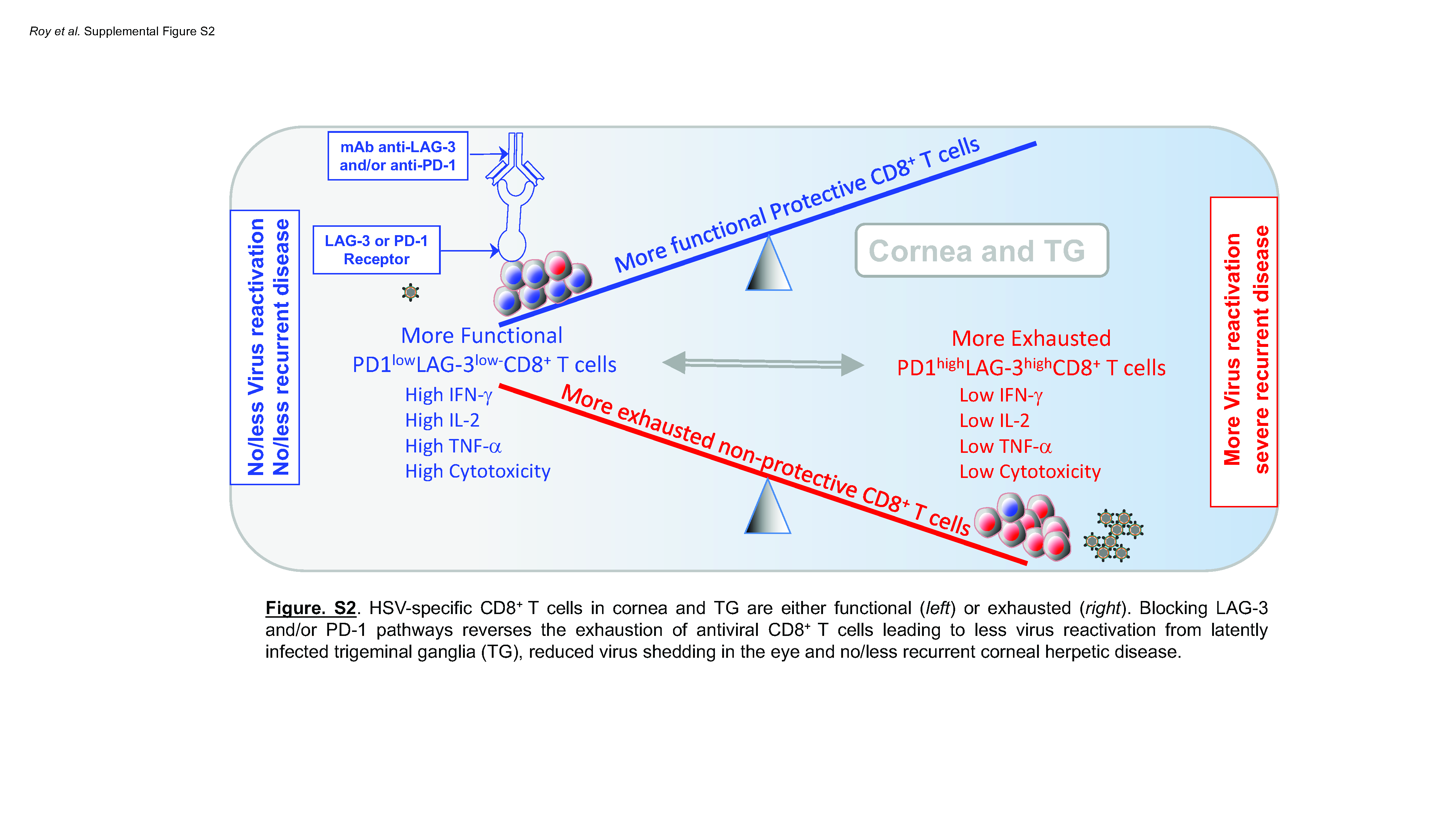

Supplement: Supplementary file 2 [file Image_2.TIFF]
